# UVC LED and Conducting Yarn-Based Heater for a Smart Germicidal Face Mask to Protect against Airborne Viruses

**DOI:** 10.3390/ma14226999

**Published:** 2021-11-18

**Authors:** Priyabrata Pattanaik, William Holderbaum, Asimananda Khandual, Hara Prasada Tripathy

**Affiliations:** 1 Semiconductor Research Laboratory, Faculty of Engineering and Technology (ITER), Siksha ‘O’ Anusandhan Deemed to be University, Bhubaneswar 751030, India; priyabratapattanaik@soa.ac.in; 2 School of Biological Science, Biomedical Engineering, University of Reading, Reading RG6 6AH, UK; w.holderbaum@reading.ac.uk; 3 Department of Textile Engineering, College of Engineering and Technology, BPUT, Bhubaneswar 751030, India; asimte@cet.edu.in

**Keywords:** healthcare, COVID-19, germicidal face mask, FAR-UV-C LED, conducting yarn-based heater, MMT, antibacterial, cytotoxicity

## Abstract

“Wear a mask. Save lives” is the slogan of WHO and all the government agencies over the world to the public. One of the most adopted prevention measures that can limit the spread of the airborne virus in the form of respiratory viral diseases, including the new strain of COVID-19, is wearing a proper mask. If the mask surface is heated to 65 to 70 °C, it could help potentially diminish any viruses or bacteria accumulated. The FAR-Ultraviolet -C (FAR-UV-C) dose for the influenza limit to 254 nm light is ~3 mJ/cm^2^/hour exposure is not harmful to the human skin and eyes. Here, we propose an intelligent mask served by FAR-UV-C and conducting a yarn-based heater that could potentially be activated in a controlled manner to kill the virus. The effective irradiation intensity for skin application would be under 0.1 µW/cm^2^. The exposure risk of UV-C is technically prevented by fabricating multi-layered fabrics with multiple functionalities. Along with experimental validation on bacterial filtration efficiency (BFE), tinker cad simulation for circuit design, and comsol multiphysics for temperature profile study, we probed Moisture Management Test (MMT) in addition with cytotoxicity risk by MTT Assay for survivability to ensure safer application potential. This novel proposed design with the germicidal combination of heating and FAR-UV-C models, described here, is promising in retaliating and combating any airborne viruses.

## 1. Introduction

An airborne virus is transmitted mainly through aerosols. It stays infectious for a more extended period and distance due to suspension in air, and causes soreness in the throat, sinuses, lungs, and nose. Such viruses include measles, chickenpox virus, mycobacterium tuberculosis, influenza virus, and, commonly, coronavirus, adenovirus, and possibly respiratory syncytial virus [1]. The worst flu pandemic in the history of the world due to its airborne nature was the Spanish flu influenza pandemic (1918–1919), caused by the H1N1 virus. A similar flu pandemic occurred in 2009 in the US, initially known as swine flu, also caused by the novel influenza virus H1N1 [2]. COVID-19 infects the human race according to three categories: symptomatic, pre-symptomatic, and asymptomatic. To reduce the rapid spread of the new strain of COVID-19, there is an urgent need to develop intelligent and efficient genocidal masks and personal protection equipment (PPE). To prevent the transmission of respiratory droplets and contact routes dominantly by mouth, eyes, nose, and hands indirectly, social distancing, frequent hand sanitation, and mask use have been advised [3,4]. N100 respirators were developed for filtering up to 99.97%. Even the omission of 0.03% confidence is a matter of grave concern. Research has been carried out on top priorities to ensure close to 100% PPE for occupational protection [5], apart from developing the vaccine. Wearable e-textiles could trigger micro-Ultraviolet-C (UV-C) LEDs [6]. Electrically conductive yarn-based heaters maintain traditional knitwear’s properties and provide comfort. The surface temperature must be within the limit for the human body, placed away from the skin, can be washable and have reusability, provide electrical insulation, etc. [7]. The physical parameters of the heating element depend upon the total amount of electrically conductive yarn row and column [8]. The International Ultraviolet Association (IUVA) believes that the correct dosing could eradicate the virus causing COVID-19, SARS-CoV-2. The UV-C light breaks down the virus’s genetic material in milliseconds [9,10,11]. Microorganisms of the size of 0.3 microns enter the UV-C Sterile Vortex (helix-shaped filter). With the help of UV-C light, it annihilates 99.9% of the continuing 5% on a DNA level, the output of which gives air that is not just clean but also medical-grade, sterile, and maintains total filtration efficiency to 99.99% [12]. Oracle Lighting proposes a new Antimicrobial Irradiation Respirator (AIR device), a wearable UVGI mask sanitizer system [13]. Conventional UV-C products use low-pressure mercury lamps with energy from the 254 nm wavelengths for their effective germicidal property. UV-C LEDs, which are innovative to the upcoming market, are generally 260 to 280 nm. The exposure risk of UV-C is technically prevented by fabricating multi-layered fabrics with multiple functionalities, while the conductive yarn layer is embedded with UV-C micro germicidal LEDs. A combination of heating and UV-C will give a more efficient and faster response [14]. Economic nano-dyeing is preferred for better comfort in common cotton fabrics, with very low cytotoxicity, moisture management properties, and a very high ultraviolet protection factor (UPF > 48). Even low GSM Nomex shirting fabric is commercially processed with UPF > 95 [15,16]. Nano Alumina (Al_2_O_3_) and Titanium dioxide (TiO_2_) are added for their antimicrobial properties and thus may inhibit bacterial growth. According to US EPA, under the EU criteria, nano Al_2_O_3_ were very toxic to the bacteria and [17]. Aluminum (Al) is the only material that has a high reflectivity for ultraviolet rays. The inner layers of fabric with a high UV protection factor will protect the user. Wearable e-textiles and tiny microprocessors trigger the conducting yarn-based heater with FAR-UV-C LED. Many research works are going on germicidal masks using either heat technology or UV-C technology individually or one after another. Using the UV-C technology in portable form with real-time use by the wearer is proposed here. This technology is already commercialized with medically approved by CDC, NIOSH, and OSHA-like organizations. In our proposed method, both technologies are applied simultaneously with tiny microprocessors with battery form in simulation environments with the theoretical calculation. Some parts were experimentally analyzed for data validation and optimization to match the existing technologies without human trial.

This piece of research work was elaborated in four segments. Motivation with a background survey of this work is in the 1st Segment, the Introduction. The technological advancement with detailed working principles, layer placement, and circuitry analysis are in Segment 2 (Section 2.1 and Section 2.2). The results of the research work with relevant discussion, safety, and efficiency of the proposed mask are in Segment 3 (Section 3.1 to Section 3.4). This research work was concluded in Segment 4 (Conclusions), followed by the Reference Section.

## 2. Technological Advancement

### 2.1. Proposed Design with Material Details

**Figure 1:**
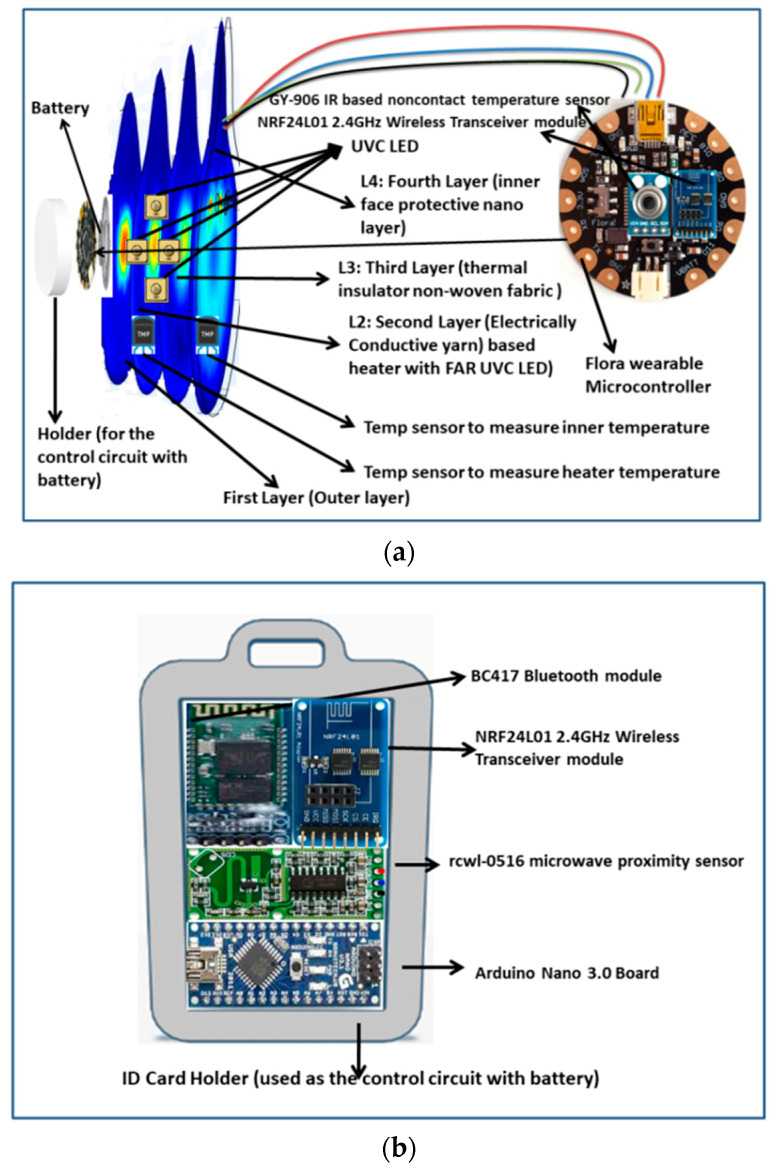
(**a**) Overall structure of the germicidal face mask for airborne virus; (**b**) identity card-based face mask control device.

The overall technological advancement structure of the germicidal mask is in Figure 1. The proposed protective mask contains four layers with height, width at mouth position, and depth is at 10 cm, 5 cm, and 6 mm, respectively. The layers of the mask have different thicknesses concerning their material composition and protection limit. They are L1, L2, L3, and L4. The outer L1 is a protective water repellent synthetic fabric, preferably polyester or nylon dyed/coated with TiO_2_ (Titanium dioxide). These fabrics were available and, after dyeing, stitched into the mask (Juki DDL-5550 Lock Stitch Industrial Sewing Machine) in the department of garment manufacturing laboratory, textile engineering department, CET, Bhubaneswar, India. We have dyed the cotton fabric with reactive dyes along with 0.5 to 3% of nano-TiO_2_, as reported in detail by khandual et al. They have superior reflecting properties, mechanical strength, stiffness, UV protection, and flame resistance.

The thickness of L1 is 0.5 mm. These will reflect again the UV emission produced by UV-C LED embedded in the L2. The high UV-C Protection Factor (UPF) above 49 with UV blocking efficiency above 98% was targeted. The UPF is well above 45 after seven times multiple washes. The L2 was made with FAR-UV-C LED for killing harmful pathogens by the action of heat and germicidal FAR-UV-C omission for the prescribed time in a controlled manner. This L2 is specifically made with cotton/polyester dyed/coated along with Al_2_O_3_ (Alumina). The heater is designed and stitched using silver plating compound yarns (SPCYs); these heating yarns are composed of a mesh of Polyester filament and micro metal conductive fibers, and are flexible. Hence, they are not affected by common washing cycles. Aluminum is the only material with high reflectivity for UV rays and has antimicrobial properties that inhibit bacterial growth. The L2 is prepared with a reflective alumina nano-dyed surface opposite of the exposure window of UV-C light. The thickness of this layer is 0.7 mm. In L3, the non-woven fabric components are used as micron-level filters for dust, microparticles, and pathogens. The thickness of the L3 layer is 4.3 mm. This L3 gives perfect thermal insulation and UV-C protection to L4 and, most notably, to face skin. The L4 is also made with cotton dyed/coated and TiO_2_ with a thickness of 0.5 mm. The specifications of this layer will be 100% cotton, plain woven scoured, bleached, and mercerized fabric with specifications: ends per Inch (EPI): 95, Picks per Inch (PPI): 70. Warp count: 38 Ne, Weft count: 33 Ne, GSM: 100 g/m^2^, Tensile strength: 28 Kgf, Elongation at break around 24 mm, bending length 130 mm with an increase in recovery angle around 75 °C.

**Figure 2:**
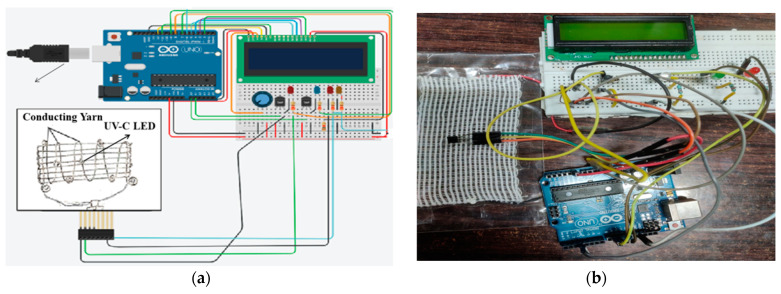

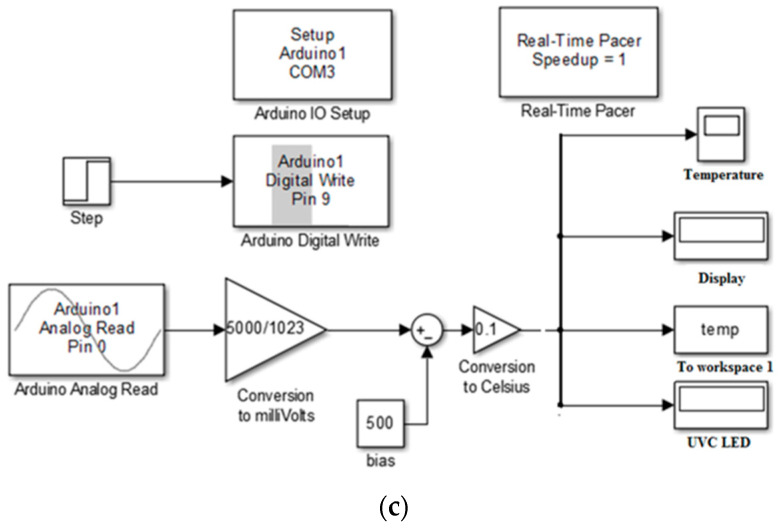
Arduino-based UV-C and conducting yarn-based heater for Germicidal Face Mask: (**a**) simulation-based circuit diagram using Tinkercad; (**b**) experimental-based circuit diagram, (**c**) Simulink block diagram for controlling output signals for conducting yarn-based heater and the FAR-UV-C LED.

An IoT-based untouchable sensor device is proposed to keep the wearer safe from airborne viruses and super spreaders. Available Safe Space device using UWB radio-based technology is one of its kind. Figure 2b depicts an identity card-based facemask controller device with microwave-based motion sensor (rcwl-0516 microwave proximity) including Arduino nano 3.0 processing board, Bluetooth module (BC-417), and 2.4 GHz wireless transceiver module (NRF24L01). For automated safety unit to keep the wearer in a safe zone. A microwave-based motion sensor is used to detect the dead zone distance between a human being up to 2 m, and the response immediately communicates to the mobile phone through a Bluetooth module. If someone reaches the dead zone, the signal will alert the wearer to maintain distance until the safe site. If someone gets a distance of 1.5 m, the IR-based non-contact temperature sensor (GY-906 IR, Gandhinagar, Gujurat, India) will activate to take the instant body temperature of the person. Above the safety zone temperature (98 °F or 36.66 °C), the face mask control device automatically switches on the UVC LED for sanitization by saying, ‘sanitization will be carried out within 5 min. Please remove the mask for safety precaution’ to the mobile device. The mask has to be removed from the face. The conducting yarn-based heater and the UVC light will be in ON state for 25 min. The heater will be ON until the temperature reaches 70 °C, then the heater will be in OFF mode and ON again if the temperature is below 65 °C. This germicidal process will be carried out in an isolated human area for the safety of the wearer.

### 2.2. Controlling Circuit Detail

The significant challenges in this proposed mask are the adverse effect of UV-C light. The heat is generated from the conducting yarn-based heater and proper controlling circuit for stable operation. To prevent the airborne virus, 100% germicidal properties of the mask are essential. Some of the methods adopted here include a precise temperature control of the heater at 70 °C for 30 min, and, at the same time, for the user to turn ON the UV-C LED for 5 min and OFF for the remaining 25 min. The proposed circuit shown in Figure 1 is takes Arduino Flora for wearable purposes. However, to view the circuit and placement before it becomes wearable, Arduino UNO has been implemented in the circuit. This is depicted in Figure 2. Two numbers of LM35 temperature sensors were implemented in different layers to sense the temperature in a precise manner. For simplicity and ease of use, a browser-based online 3D modeling program called Tinkercad was used to construct and test the circuit before implementation.Figure 2a depicts the proposed circuit design, whereas Figure 2b shows the practical model of the design with a fabric heating pad and 260 nm UV-C LED.

For primary design, 16 × 2 LCD was implemented to visualize the exact temperature of both the heater (L2) and face (L4). LM35 senses the temperature value, and their analog proportional output data values are fed to Arduino analog data output pins of A0 and A1. The microprocessor programming then converts the analog values into temperature data values. These data values are again compared with prescribed set points to simulate the programming and experimental operation smoothly. The set points are 65 °C to 70 °C for L2 and 20 °C to 35 °C for L4. The L2 temperature was maintained between 65 °C to 70 °C to annihilate the airborne virus. Additionally, the L4 temperature was maintained between 20 °C to 35 °C for comfortable use of a mask for a more extended period from the inner side.

Programming is carried out to maintain the set point accurately. The fluctuation in the temperature values from their desired set point values creates a response signal which turns the heater either ON or OFF depending upon the lower or higher set point values. In the outer heater temperature, the heater turns OFF if the temperature value rises above 65 °C. The corresponding LED, which has connected in parallel with the heater, can visualize the ON and OFF mode of the heater. The turning ON of the UV-C LED depends on the heater temperature as the set point value of 70 °C energizes the UV-C LED and below the set point, thus turning the UV-C LED OFF. In the proposed model, FAR-UV-C LED light with a wavelength of 222 nm has preferably been used for better germicidal properties. The real-time sensing data will be extracted from Arduino UNO with the help of Simulink. It has been implemented to accurately track the set point values by implementing a biasing deal to the model precisely. This helps the programmer model a specific piece of work independently from Arduino, an open-source electronics platform. The live link data values from sensors work and are controlled by the Simulink model alone without giving any extra effort. The detailed Simulink model is depicted in Figure 2c. In Figure 2c, four output blocks are displayed. They are designed in such a way that they only give accurate conversion outputs from the modeling.

According to International Commission on Non-Ionizing Radiation Protection (ICNIRP), the UV-C dose for coronavirus and other seasonal viruses such as influenza limit to 254 nm light with the dose is 23 mJ/cm^2^ per 8 h or ~3 mJ/cm^2^/h exposure. Continuous FAR-UV-C exposures at this intensity will result in 90% viral inactivation in around 8 min. A total of 95% viral inactivation is achieved in almost 11 min, 99% inactivation in about 16 min, and 99.9% inactivation in about 25 min. Increasing the intensity by two will result in these disinfection times while maintaining safety [18]. The major apprehension from industry experts about FAR-UVC is inadequate testing on humans. During trials on mice, it was proved with no long-term side effects. Therefore, there are restrictions to the quantity of UV energy in a space. This requires FAR-UV-C to drive at an extremely low dose over a lengthy period. The FAR-UVC technology is new to the market. Due to the difficulty in manufacturing efficient FAR-UVC LEDs and the limitation of the fabrication process with the industry-standard epitaxial techniques, there is an inadequate product available to use. Therefore, we are experimenting with commercially available 260 nm UV-C LED with a dose of 8 mJ/cm^2^ for a 5 min duration. The entire circuit is assembled in a ring-type holder with a battery case, as shown in Figure 1, and placed with the ventilation cap of the mask.

## 3. Results

**Figure 3:**
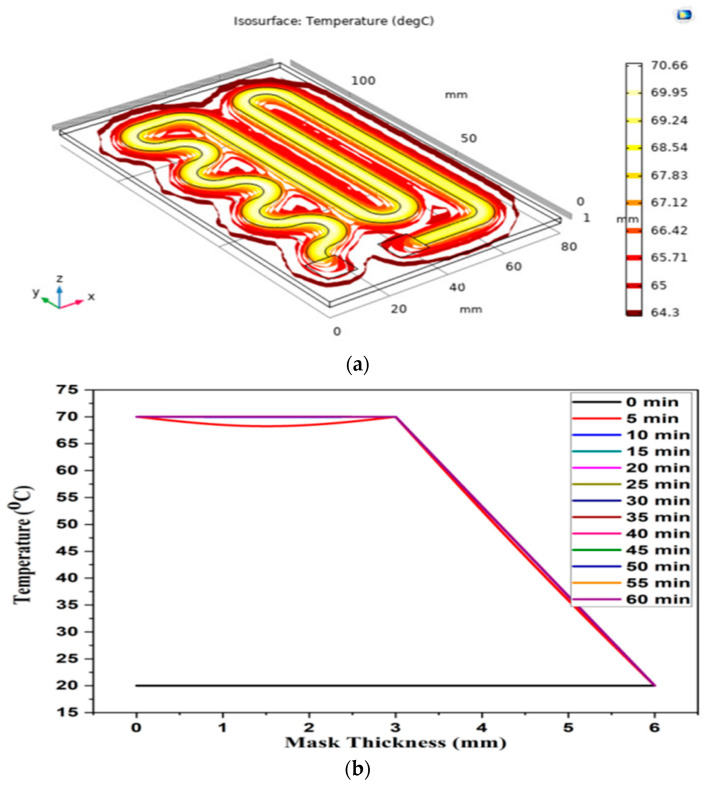
(**a**) Temperature profile of the mask at outer surface at L1; (**b**) total temperature with respect to the thickness of the mask.

**Figure 4:**
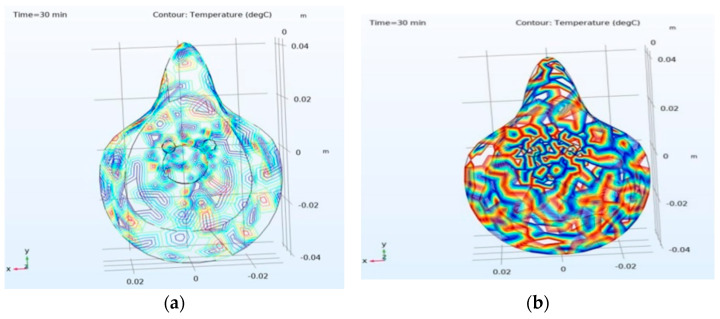
Temperature profile of the mask: (**a**) Inner layer at 20 °C; (**b**) outer layer at 70 °C.

In the experimental setup, the 260 nm UV-C LED with 8 mJ/cm^2^ dose is set to glow for 5 min ON time and 10 min OFF time and repeat the process until the heater is in OFF mode or the circuit is in shutdown mode. Figure 3 analyzes the temperature profile of the outer layer surface and the change in temperature of the mask at different layers with the conducting yarn-based heater. The face of the heating element is towards the outer layer of the mask and the temperature varying from 70 °C to 64.3 °C on the surface of L2 as shown in (Figure 3a). However, the fabric temperature drops significantly from 65 °C to 35 °C at L3 of the mask and further drops from 35 °C to 20 °C in L4 as shown in (Figure 4). Figure 4 depicts the entire mask temperature profile at both inner layer (Figure 4a) and outer layer (Figure 4b), respectively. For the duration of 30 min and 20 °C inner mask temperature, water vapor saturation pressure and the moisture content is 2.34 × 10^3^ Pa and 7.25 × 10^−3^, respectively. Similarly, at same time duration and 70 °C outer temperature, the water vapor saturation pressure and moisture content are 3.12 × 10^4^ Pa and 0.11 Pa, respectively for a 30 min duration in a comsol simulation environment as the simulation platform gives a perfect track of temperature at each layer. It also enables researchers/manufacturers to design an ideal layered mask with proper thickness. For 30 min with 20 °C inner mask temperature, water vapor saturation pressure and the water content is 2.34 × 10^3^ Pa and 73%, respectively. Similarly, at the same time duration and 70 °C extreme temperature, the water vapor saturation pressure and water contents are 3.12 × 10^4^ Pa and 11%, respectively. The MMT study also noted that the water content in the Inner layer was 19% with a wetting time of 8.938 s and 96% with a wetting time of 119.953 s, respectively.

(0.018 P)/(0.029 (P − p)) = ((0.62 p))/((P − p)))(1)

**Figure 5:**
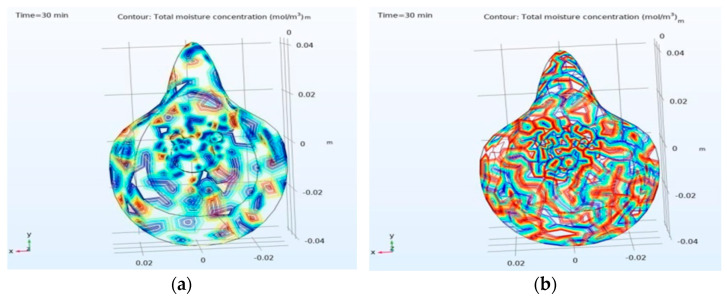
Total moisture concentration of the mask: (**a**) inner layer is 0.48 mol/m^3^; (**b**) outer layer is 5.47 mol/m^3^.

Equation (1) found in [19,20], the total atmospheric pressure, water vapor pressure, and room temperature pressure variation are denoted as P, p, and (P − p), respectively. At room temperature, the (P − p) value is nearly equal to P ~10^5^ Pa. As per the mathematical calculation, the moisture concentration in the air is the function of (0.62 × 10^5^ P)/~10^5^. By applying the values in Equation (1), the calculated and simulated moisture concentration obtained 0.62 mol/m^3^ and 0.48 mol/m^3^ for the inner layer and 7.75 mol/m^3^ and 5.47 mol/m^3^, respectively, for the outer layer. Figure 5 depicts the above elaboration both for inner and outer mask moisture concentration.

### 3.1. Safety and Efficiency of the Proposed Mask with Respect to Temperature

The L3 made up of non-woven material. Thus, L3 layer acts as a transient thermal barrier by regulating the heat radiated from the heater layer to the inner layer L4 of the proposed mask. This helps the wearer to wear the mask in any adverse environmental situation [20].

### 3.2. Safety and Efficiency of the Proposed with Respect to UV-C LED

UV-C LED in between 180–270 nm is germicidal. From the experimental procedure and ICC-RTqPCR test on HCoV-OC43, it is observed that 260 nm UV-C with exposure of 8 mJ/cm^2^/h would have a 99% effect for viral inactivation in ~5 min. A low dosage of 2 mJ/cm^2^ of 222 nm UV-C LED has the efficiency to annihilate the airborne influenza virus (H1N1), whereas dosages at 1.7 mJ/cm^2^ and 1.2 mJ/cm^2^ the human coronavirus from group alpha (HCoV-229E) and beta (HCoV-OC43) are inactive. For the continuous FAR-UV-C exposure of 3 mJ/cm^2^/h recommended for viral inactivation of 99.99% in ~25 min [18]. In this case, the effective irradiance of UV-C device is weighted against the effective spectral peak curve of 260 nm. The effective irradiance effect of the UV-C device was calculated from Equation (2) [18,19,20]. The spectral irradiance, relative effectiveness, and bandwidth are denoted as *E_λ_*, *S*(*λ*), and ∆(*λ*), respectively.

(2) $$E_{eff}=\int_{180\;\mathrm{nm}}^{270\;\mathrm{nm}}E_{\lambda }\;S\left(\lambda \right)\;∆\left(\lambda \right)\;$$

For the surface disinfection applications, the UV dosage is the product of irradiance and their exposure time. Equation (3) denotes the UV dosages for surface disinfection [21].

D = E_λ.t(3)

where D (mJ/cm^2^) is the dose, E_λ. (µW/cm^2^) is the irradiance, and t is time in seconds. From Equation (3), one can easily calculate the spectral irradiance and relative effectiveness of UV-C dose. The lowest dose for HCoV-229E and HCoV-OC43 are 1.7 mJ/cm^2^ and 1.2 mJ/cm^2^, respectively, with irradiance value 0.13 µW/cm^2^ and 0.08 µW/cm^2^. The effective and safe irradiation intensity for the skin application should not be increased above 0.1 µW/cm^2^. The peak intensity limit to 0.5 µW/cm^2^ was suggested for irradiation time of 8 h/day [20,22,23]. Hence, the use of FAR-UV-C LED is safer for a wearer with a continuous radiation up to 25 min to inactivate the aerosolized alpha coronavirus 229 E and beta coronavirus OC43 to the extent of 99.99%.

### 3.3. Bacterial Filtration Efficiency (BFE)

Common filtration efficiency was checked in the range of 1–5-micron aerosol sizes [24] as it covers the majority of human-viable infectious viruses in aerosols. We have conducted the ASTM F2101-19 Standard Test Method for Evaluating the Medical Face Mask Materials, Using a Biological Aerosol of Staphylococcus aureus. The mean of three readings was found to be 96.25. The conditions are as follows.

ASTM F2101-19:

Biological Aerosol: Staphylococcus aureus;
Area contacting with the bacterial Challenge: Inside the mask;
Mean particle size of the challenge Aerosol: 3 µm ± 0.3 µm;
Test Area: 50 cm^2^ Approx.

### 3.4. Moisture Management Tests (MMT), Antibacterial and Cytotoxicity Analysis

The moisture management tests were carried out in MMT, SDL atlas. The improved moisture management properties are seen in the case of TiO_2_, followed by Al_2_O_3_. It establishes a significant variation in water penetration levels between controlled and treated samples. The antibacterial effect on clothes embedded with TiO_2_ and Al_2_O_3_ nanoparticles dyed with different bacterial strains is considered. No inhibition zone was found with clothes dyed with 1% and 3% nanoparticles in a controlled environment. It happens due to nanoparticles being firmly attached to the fibers of the clothes. It is also physically interpreted. To study the cytotoxicity, TiO_2_ and Al_2_O_3_ nanoparticles were dyed at various concentration levels: 1%, 2%, and 3% shades of the fabrics with reactive remazol dyes, along with nanoparticles concentration ranges (0.01%, 0.02%, and 0.03% weight of the fabric) [15,16,24]. The modification in morphology and their effect on the viability of skin cell lines are analyzed. The human squamous epithelial cell line A431 cells were cultured in Dulbecco’s Modified Eagle Medium supplemented with 10% fetal bovine serum, 2 g/L sodium bicarbonate, and 2 mm l-glutamine. A431 cells were cultured with 5% CO_2_ in an air atmosphere at 37 °C in a humidified incubator. A431 cells showed their survivability in order TiO_2_ > Al_2_O_3_ at all impregnation concentrations by exposing the cells for 24 h. The MTT assay results supported the morphological analysis where the cell viability decreased to 86% and 55% at 0.03% impregnation of TiO_2_ and Al_2_O_3_ nanoparticles, respectively [16]. MTT assay for survivability was conducted again for 0.01%, 0.02%, and 0.03% Nano- TiO_2_ and Al_2_O_3_-treated cotton, and three sample means and the standard deviation were given in Table 1 below. Hence, 0.01%, 0.02% levels of TiO_2_-treated cotton would be safer in application in terms of cytotoxicity. The degree was in order TiO_2_ > Al_2_O_3_. The absorbance was taken at 570 nm in an enzyme-linked immune sorbent assay (Elisa) plate reader (Epoch, Biotek, Germany). Additionally, the feasibility of the cells decreases by 10% at a lower percentage of nanoparticles. The moisture management tester concludes that the TiO_2_-treated fabric is a moisture management fabric with a minimal cytotoxicity risk [15].

## 4. Conclusions

Sooner or later, a newly designed future innovative mask will likely come about. The situation demands that it must kill any fatal viruses, and not just protect against them. We proposed here a new concept with a theoretical and experimental background: the FAR-UV-C light, being one of the most established techniques for quicker and safer disinfection. Most importantly, the FAR-UV-C-LED irradiation efficiently eradicates pathogens without harming the human cells or tissue. A low dose of 2 mJ/cm^2^ of 254 nm FAR-UV-C-LED has the efficiency to annihilate airborne influenza virus (H1N1), and human coronaviruses from group alpha (HCoV-229E) and beta (HCoV-OC43). They seem promising with the sciences of textile materials, which could ensure safety with stability in the long run. The experimental validation on bacterial filtration efficiency for three microns was found to be 96.25%. The proposed design was conceptualized and explained in thinker cad. The heat generation profile by using the conducting yarn-based heater was analyzed in the comol multiphysics model. TiO_2_ nano-dyed fabric layers were designed to ensure a high UV protection factor; outer and inner layer UPF were found to be above 49 and the UV blocking efficiency above 98%. The Moisture Management Test (MMT) and cytotoxicity risk by MTT Assay for survivability ensure safer application potential using TiO_2_ at 2% shade, not Al2O3. This novel proposed design with the germicidal combination of heating and FAR-UV-C models, described here, is promising in retaliating and combating any airborne viruses. It could have a synchronous effect of both far UV-C and heat; about 70 °C could be attended to quickly, and controlled as per the user’s wish. Interestingly, the IoT enabling technologies, intelligent control, and research would be augmented too in this proposed design.

## Figures and Tables

**Table 1:** MTT assay for survivability: 0.01%, 0.02%, and 0.03% Nano- TiO_2_ and Al_2_O_3_-treated cotton.

	TiO_2_			Average	Stdev.
Control	100.00	100.00	100.00	100.00	0.00
0.01	98.14	98.20	96.82	97.72	0.78
0.02	92.12	86.56	88.14	88.94	2.87
0.03	87.34	86.63	83.78	86.00	1.88
	**Al_2_O_3_**				
Control	100.00	100.00	100.00	100.00	0.00
0.01	69.31	66.67	64.90	66.96	2.22
0.02	61.90	65.31	62.82	63.34	1.76
0.03	44.71	55.85	54.97	51.84	6.19

## Data Availability

Data sharing not applicable.
